# Spatial Distribution of Hospitalizations for Ischemic Heart Diseases in the Central Region of Asturias, Spain

**DOI:** 10.3390/ijerph182312320

**Published:** 2021-11-24

**Authors:** Isabel Martínez-Pérez, Verónica González-Iglesias, Valentín Rodríguez Suárez, Ana Fernández-Somoano

**Affiliations:** 1 IUOPA-Área de Medicina Preventiva y Salud Pública, Departamento de Medicina, Universidad de Oviedo, C/Julián Clavería s/n, 33006 Oviedo, Spain; gonzaleziveronica@uniovi.es (V.G.-I.); fernandezsana@uniovi.es (A.F.-S.); 2 Dirección General de Salud Pública, Consejería de Salud, Principado de Asturias, C/Ciriaco Miguel Vigil, 9, 33006 Oviedo, Spain; valentin.rodriguezsuarez@asturias.org; 3 CIBER Epidemiología y Salud Pública (CIBERESP)—Instituto de Salud Carlos III, Monforte de Lemos Avenue, 3-5, 28029 Madrid, Spain; 4 Instituto de Investigación Sanitaria del Principado de Asturias (ISPA), Roma Avenue s/n, 33001 Oviedo, Spain

**Keywords:** acute myocardial infarction, angina pectoris, hospital admission, spatial distribution, disease mapping

## Abstract

Hospitalizations for ischemic heart disease have an uneven distribution throughout Spain, in which Asturias is the community with the highest rates of acute myocardial infarction (AMI) and angina pectoris (AP). Cardiovascular diseases are related to environmental, socioeconomic and previous medical conditions, which result in geographical differences in the incidence of hospital admissions and mortality. The goal of this study was to describe the spatial distribution of hospital admissions in the central area of Asturias and explore the existence of spatial patterns or clusters. Urgent hospital admissions for AMI and angina AP in the hospitals of the central area of Asturias were registered, geocoded and grouped by census tracts. Standardized admission ratio, smoothed relative risk, posterior risk probability and analysis of spatial clusters between relative risks throughout the study area were calculated and mapped. Geographical differences were found in the distribution of hospital admissions for AMI and AP across the area and between men and women. The cluster analysis indicated contiguous census tracts with high relative risk values in the northwest region of the study area and low relative risk in the east, particularly for men. The geographical analysis shows the existence of patterns and spatial clusters in the incidence of AMI and AP, for both men and women, in the central area of Asturias.

## 1. Introduction

Cardiovascular diseases are the leading cause of death worldwide [1]. It is estimated that they cause 17 million deaths annually, of which approximately 7.4 million correspond to coronary heart diseases. In the European Union, cardiovascular diseases had a mortality rate of 63 cases per 100,000 inhabitants in 2017 [2]. However, there are large geographical differences throughout Europe, with Spain being one of the countries having a lower rate, i.e., 37 cases per 100,000 inhabitants. In 2019, there were 29,247 deaths from ischemic heart diseases registered in Spain (International Classification of Diseases—ICD10 I20-I25), of which 13,673 were caused by acute myocardial infarction (AMI) (ICD10 I21), and 13,371 by chronic ischemic heart disease (ICD10 I25) [3]. Diseases of the circulatory system also cause a high percentage of hospital admissions, reaching 12.5% in 2018 in Spain, second only behind respiratory diseases (13.0%). The data published in Spain for 2018 included 118,464 hospitalizations for ischemic heart diseases, which represented 2.4% of total hospitalizations, and 19.4% among all circulatory diseases. The rate of hospitalizations for ischemic diseases was unevenly distributed in the different autonomous communities, with Asturias being the community with the highest rate both for angina pectoris (AP) (57 cases per 100,000 inhabitants) and for AMI (158 cases per 100,000 inhabitants). Both of these rates are above the national average of 31 and 126, respectively, according to the data from 2018 [4].

Geographical information systems and spatial analysis techniques focused on epidemiology can help analyze the spatial variable in the geographical context of a disease, so that the analysis can be focused of the spatial distribution of social risk factors and environmental factors that influence health, the existence of geographical trends, and associations or clusters and their influence on the distribution of the diseases [5].

The existence of inequalities in the geographical distribution of diseases plays an important role both for etiological research and for public health management services. In this manner, specifically targeted follow-up campaigns can be created to detect the existence of deficiencies or shortcomings in care networks. Moreover, measures directed at areas with higher incidence rates of the diseases can be implemented to mitigate environmental or genetic background effects [6,7].

The distribution of the population in Asturias exhibits a clear asymmetry, with a very dense central zone, in comparison to the regional borders. The triangle formed by the three main cities (Avilés, Gijón and Oviedo) concentrates almost 70% of the population of the Autonomous Community in a territory of about 1000 km^2^, i.e., approximately 10% of the area of the region, which could be seen as a homogenous territory. Thus, the goal of the present study was to determine the distribution of hospital admissions for AMI and AP in the central area of Asturias, in addition to trends and/or spatial patterns.

## 2. Materials and Methods

### 2.1. Study Area and Reference Population

**Figure 1:**
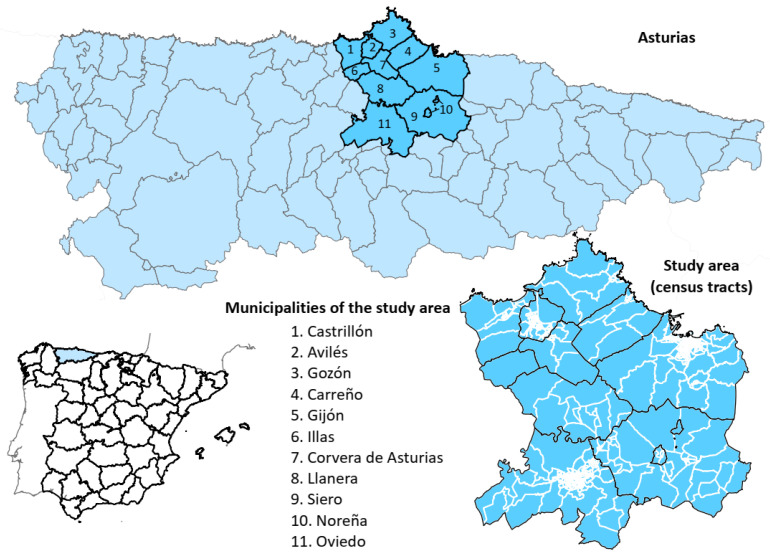
Location of the study area. Asturias is an Autonomous Community of Spain, located in the north of the country. The study area is comprised of eleven municipalities in the central area of the region.

The study area (Figure 1) comprises eleven municipalities in the central area of the Autonomous Community of the Principality of Asturias, with a total population of 705,968 inhabitants. The 2016 Municipal Register of Inhabitants was used as a reference value. It was the first year of the study sample, which was obtained from SADEI (Asturias Society of Economic and Industrial Studies). The individuals were categorized by age groups (less than 15; 15 to 39; 40 to 64; 65 to 84; 85 years and over) and sex. The study population corresponded to 67% of the total population of the region (1,042,628 inhabitants in 2016). The four municipalities with more than 50,000 inhabitants in the Autonomous Community were included (Avilés, Gijón, Oviedo and Siero), thus comprising 81.32% of the study population.

### 2.2. Study Population

The health data analyzed corresponded to unscheduled (urgent) hospital admissions in the hospitals of Avilés (San Agustin University Hospital), Gijón (Cabueñes University Hospital and Jove Hospital) and Oviedo (Asturias Central University Hospital). The study focused on hospital incidence of admissions for AMI and AP. The data were obtained from the Specialised Care Activity Registry RAE-CMBD—Minimum Basic Data Set, corresponding to the period 2016–2018, recorded according to the ICD code (ICD10: I-20—I-21).

In this registry, each entry corresponded to an admission event, and included a personal identifier for each registry, sex, date of birth, date of admission, and main diagnosis. Through the personal identifier, the residence addresses entered in the SIPRES (Population and Health Resources Identification System) were obtained for their geographical allocations. The ICD10: I20-I21 records were taken for all age groups (less than 15, 15 to 39, 40 to 64, 65 to 84, 85 and over).

### 2.3. Population Area and Reference Cartography

The unit of study was composed of the census tracts (CTs) of the municipalities under study (558 CTs), obtained from the National Institute of Statistics. The CTs are regulated by current legislation [8] and are used in medical, economic, and sociological studies as they are the minimum population spatial unit and maintain homogenous population data across the country. Their total reference population was determined through the code of the CT contained in the dataset of the Municipal Register of Inhabitants. The CT constituted the most homogeneous units in terms of population, with an average of 1265 inhabitants for the 558 CTs included. The population density of the area of study depends, also, on the size of each CT, which is high in the central areas of the major cities of the study, and low in the rural areas, as characterized by its population dispersion (Figure S1).

### 2.4. Procedure

The health registry data was geocoded according to the portal level, using the addresses entered in each health event. Geocoding was performed using ArcGis 10.4, based on the cartographies of specific portals of the city councils of Avilés, Gijón and Oviedo, and the cartography of the project ‘CartoCiudad’ of the National Geographic Institute for the rest of the municipalities. Once the health events were geocoded, they were grouped by CT, disease, sex, and age group. Analysis were run independently for each sex, men and women, taking into account the differences in risk factors, incidence and diagnosis of AMI and AP between each sex [9,10].

For each geographical unit (CT), we calculated the standardized admission ratio (SAR), i.e., the quotient between the numbers of observed and expected admissions. The expected admissions for the group of municipalities in the study area was calculated for each sex, using the indirect method of standardization and the specific rates for age groups in the study period of the central area considered.

Due to the variability of the SAR, resulting from areas with small population size or with infrequent health events, it was considered necessary to apply spatial smoothing methods. To that end, the smoothed relative risk (SRR) of admission for AMI and AP was calculated using conditional autoregressive models developed by Besag, York and Mollié [11]. The models were run using the BYM [11,12] prior and compared with iCAR [13] and Leroux [14] priors to determine which model would be more accurate for the study area. These are spatial Poisson models with random effects that take into account the spatial adjacency of the geographical units of the area. Their use is simplified using the Laplace approximation technique to perform Bayesian inference, following the integrated nested Laplace approximation procedure [15,16]. Both SAR and SRR are expressed in percentages. In order to determine the better fitted model, the deviance information criterion (DIC) [17] and Watanabe–Akaike information criterion (WAIC) [18] were calculated for each model.

In addition, the posterior risk probability (PP) was calculated, i.e., the probability that the smoothed risk was greater than 100. A PP value ≥0.8 indicated a statistically significant admissions excess (not due to chance).

The Stata v14 and R version 3.6.1 programs were used with the INLA library (R-INLA Project) for calculating the SAR, SRR, and PP.

Multivariate analysis models were also derived, using the INLAMSM R package [19], in order to take into account the data of both men and women to estimate risk in the area of study and compare them with the independent analysis.

The analysis of spatial clusters was performed using Moran’s, index, which measures the spatial autocorrelation between the smoothed relative risks throughout the study area, and tries to contrast the null hypothesis of the absence of global spatial autocorrelation (i.e., spatial randomness) versus the alternative hypothesis of the existence of spatial autocorrelation.

In a complementary manner, we calculated local indicators for the purpose of detecting a possible spatial autocorrelation in a certain subset of spatial units. In this way, an index could be obtained for each spatial unit studied, which made it possible to assess the degree of individual dependence of each spatial unit with respect to the others. To that end, we used the local Moran statistic, proposed by Anselin [20], whose interpretation is similar to that of Moran’s index, i.e., if it is statistically significant and positive, it confirms the presence of a cluster of similar values around the spatial unit ‘i’. On the contrary, if it is statistically significant, but negative, there will be a cluster of different values around the nth spatial unit (spatial outliers). The results of spatial autocorrelation at the local level are presented using the local indicators of spatial association (LISA), which used the local Moran’s indices calculated for all the assessed spatial units (CTs), allowing the geographical determination of (1) spatial groupings, which occur when a spatial unit that registers a high/low value of the variable is surrounded by spatial units that also register high/low values of that variable, i.e., high–high or low–low; and (2) spatial outliers, which arise when a spatial unit with a high value of the assessed variable is surrounded by spatial units in which the variable registers small values or vice versa, i.e., high–low or low–high.

## 3. Results

The geocoded and analyzed health events amounted to 3218 of a total of 3251 contained in the original database, representing 98.99%. Some records (1.01%) were excluded due to the impossibility of spatial determination through the addresses entered. The records corresponded to men (64.42%) and women (35.58%).

The model comparison using different priors showed that the BYM prior gave the best adjustment to the model, both in men and women, and was used to map the results.

**Figure 2:**
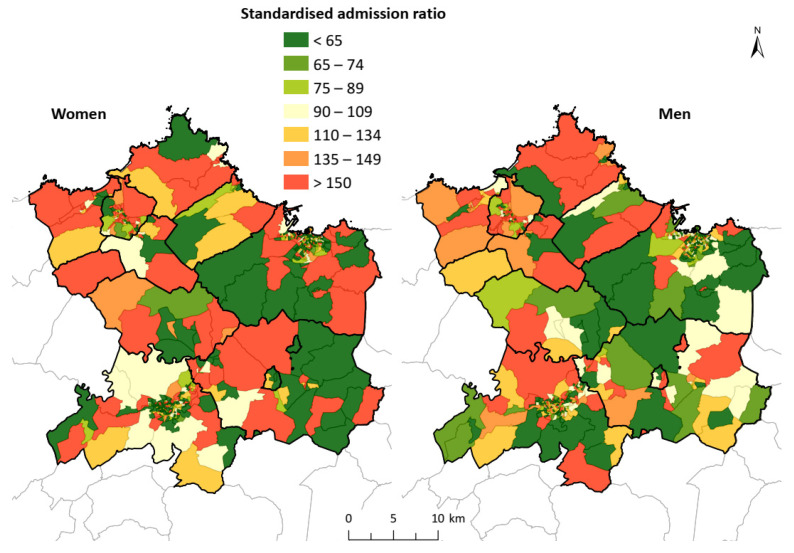
Standardized admission ratio for AMI and AP in the study area. The geographical distribution of the standardized admission ratio shows a dispersed distribution of high values for both men and women, without a clear pattern in the distribution.

The geographical analysis of the SAR (Figure 2) indicated a dispersed distribution of values above 100 for men, although more concentrated in the north and northwest of the study area. In the case of women, the distribution was similar, without a clear pattern in the distribution; however, there were CTs with high SAR values observed in the central zone of the study area. For men, 43.73% of the CTs was above a value of 100, and this value was 43.37% in the case of women.

**Figure 3:**
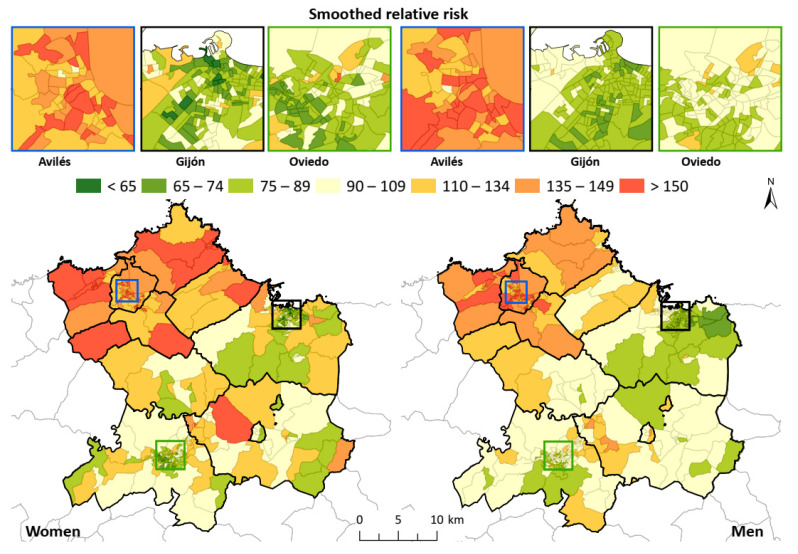
Smoothed relative risk for AMI and AP in the study area. For both men and women, the concentration of values was above 100 in the northwest of the study area, which corresponded to the area of Avilés and neighboring municipalities, reaching the west of Gijón. Oviedo and Gijón center areas show lower values across the cities compared with Avilés.

The representation of the smoothed relative risks (Figure 3) smoothed the standardized admission ratio, thus allowing better spatial analysis of the underlying patterns. For both men and women, the concentration of values was above 100 in the northwest of the study area, which corresponded to the area of Avilés and neighboring municipalities, reaching the west of Gijón. Furthermore, in both men and women―especially in the latter―a homogeneous pattern of high risk could also be observed in the central zone, between the municipalities of Siero and Oviedo. This is included in the magnified images over the biggest cities included in the area (Avilés, Gijón and Oviedo) in order to have a better understanding of the result in the most crowded areas.

**Figure 4:**
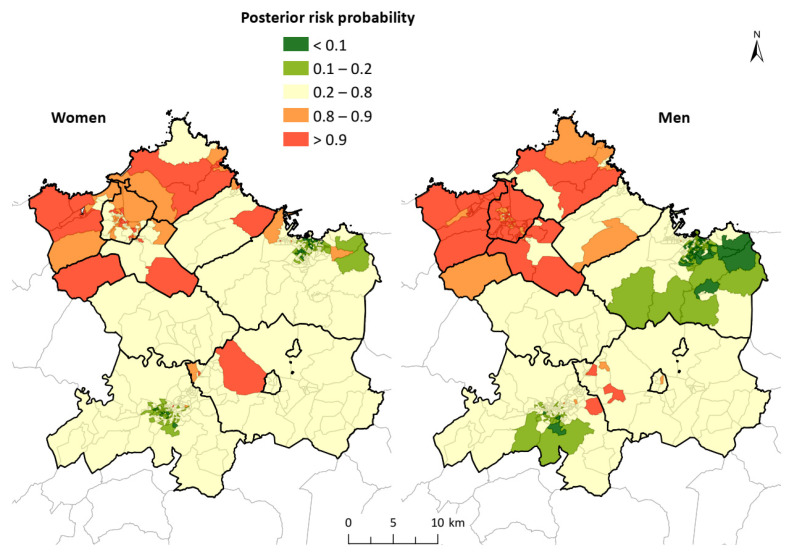
Posterior risk probability for AMI and AP in the study area. The spatial distribution of the PP indicated how the northwest zone contained numerous CTs in which the values were greater than 0.8; this result was similar for men and women.

The spatial distribution of the PP (Figure 4) indicated how the northwest zone contained numerous CTs in which the values were greater than 0.8, which was similar for men and women. In addition, specific census tracks were observed inside the study area (surroundings of Oviedo and Siero), where these values were also exceeded, confirming the patterns observed in the SRR map. Overall, 19% of the CTs regarding men, and 14% considering women, exceeded the value of 0.8. It is also worth noting the existence of a wide area of values below 0.2 (low probability of risk) to the east and south of Gijón―more evident in men―and to the south and in the center of Oviedo.

**Figure 5:**
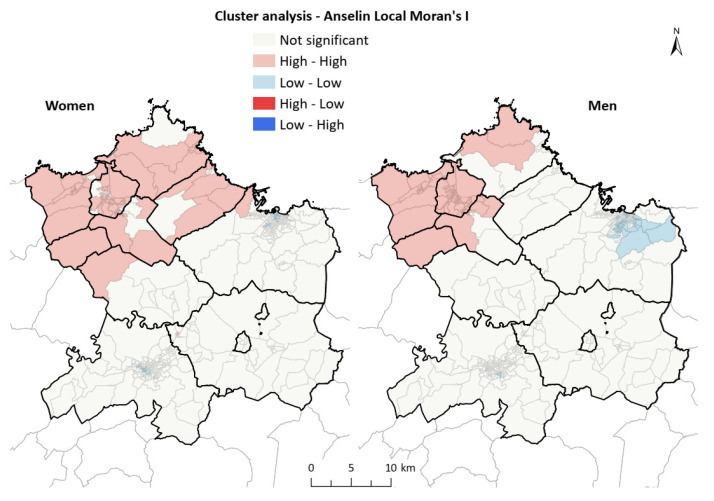
Cluster analysis (LISA) of AMI and AP in the study area. There were high-value grouping areas in the Avilés region (councils of Avilés, Castrillón, Corvera de Asturias, Illas and Gozón) in the case of men, extending to Carreño and Gijón for women. In the case of men, the presence of a group of low values in the central and eastern areas of Gijón was notable.

The correlation between SAR values of men and women, although low, was positive (Pearson 0.1735) and the multivariate models showed a clear geographic pattern similar to those obtained independently for men and women. The northwest area showed high values, above 100 for RRS, and 0.8 for PP, whereas the south and east had low values in the rates of both men and women. This analysis highlights the presence of CT with a statistically significant excess of hospital admissions in the central area, corresponding to Siero and Oviedo. This was more obvious in men, and also remarkable in women, but was not so clear in the independent analysis (see complementary Figures S2 and S3). The analysis of spatial correlation (Moran’s index) indicated that, for both men and women, there was a statistically significant spatial aggregation: Iwomen = 0.638 (z = 26.3); Imen = 0.838 (z = 34.2). The analysis of LISA, by comparison (Figure 5), indicated the areas where the high and low values of incidence of the disease were grouped, making it clear that there were high-value grouping areas in the Avilés region (councils of Avilés, Castrillón, Corvera de Asturias, Illas and Gozón) in the case of men, and extending to Carreño and Gijón for women. In the case of men, the presence of a group of low values in the central and eastern areas of Gijón was notable; however, it was not indicated by the analysis of women, where only some CTs were observed in the center of Oviedo and Gijón under this aggregation.

## 4. Discussion

The results of the present study indicated the existence of a clear geographical differentiation in the incidence of hospital admission for AMI and AP in the central area of Asturias. We assessed small geographical units, in this case, CTs. The standardized admission ratios assessed indicated a relatively heterogeneous pattern for both men and women. However, a north–south trend could be observed in the grouping of high values, which could be seen to be more clearly representing the distribution of SRR, eliminating a certain relative variability and taking into account spatial adjacency. The maps of SRR illustrate a grouping of high values in the CTs located in the northwest, which was more concentrated in men; whereas, in women, there was also an aggregation of high values in the central zone of the area, in addition to in the aforementioned northwest zone. It is worth noting than the city centers of both Gijón and Oviedo, the biggest cities of the study area, have almost no values above 100, whereas Avilés has almost all the CTs with higher values. The analysis of clusters and spatial aggregation more clearly described the situation, because the high values were grouped in the CTs of Avilés, Castrillón, Carreño and Gozón, reaching Carreño and the western area of Gijón in the case of women, and also showing a statistically significant correlation in the study area with respect to both sexes. The multivariate analysis undertaken to account for the correlations between both sexes showed a similar pattern in PP as those shown in the independent analysis. This provided a clear view of the high values of the area of Avilés for both sexes, and a more complex insight into the central area (Oviedo and Siero), especially in men, with higher values in a larger area than previously observed.

The area of study is an industrial environment with a dense population. The main cities and industrial areas of the region are structured―especially those next to the estuaries of Avilés (municipalities of Avilés, Corvera de Asturias, Gozón and Castrillón) and Aboño (between the municipalities of Gijón and Carreño), and the port of El Musel (Gijón)—to take advantage of the accesses by sea. Moreover, there are cities and industrial areas in the central regions of the municipalities of Llanera and Siero that benefit from the availability of land and good road accesses [21,22].

By comparison, it has been documented that, in hospitals of the area, there was a positive association between unscheduled admissions for AMI and AP in the 2003–2018 study period and the daily mean levels of atmospheric pollutants, especially SO_2_. Moreover, the association with PM and NO_2_ was also statistically positive. It is worth noting the association found in Avilés Hospital, which is located in the aforementioned northwest region of the study area, where the cumulative incidence of hospital admissions for AMI and AP was clearly higher than in the remainder of the municipalities of the area, and one of the highest observed among the Spanish health areas as a whole [23].

A consistent number of published scientific studies addressing the spatial distribution of heart disease morbidity have highlighted health inequalities between different geographical areas, similar to the results obtained in the present study. In France, in the Etang-de-Berre region, an excess risk of hospitalizations due to myocardial infarction was detected for both men and women living in districts exposed to atmospheric pollution of industrial origin [24]. By comparison, in Denmark, a more complex pattern was found by studies that assessed the incidence of AMI and social inequalities that are not only explained by sociodemographic differences, but also point to other determinants, such as geographical inequalities, deprivation or exposure to environmental pollutants [25]. This outcome is similar to that in the Municipality of Madrid, according to a study that addressed mortality from cardiovascular diseases [26]. In addition, some studies have highlighted the impact that traffic and port activities have on air quality, in addition to the exposure of the populations to pollutants in urban areas near ports [27], which may also be influencing the central area of Asturias and thus should be analyzed.

The geographical differences found in the distribution of hospital admissions for AMI and AP in the central area of Asturias, in terms of the reasons for admission, the SRR, and the probability of subsequent risk, for both men and women, indicated the existence of CTs with high risk values, concentrated in the north and northwest of the study area, and especially in the Avilés region. The cluster analysis at the local level (LISA) indicated, for both sexes, the existence of aggregates of CTs with high relative risk values in the northwest region of the study area, revealing the existence of underlying causes that may be enhancing the risks of admission for heart diseases in this area. This finding should be assessed in detail.

Studies based on spatial inequalities in the incidence of diseases, in this case AMI and AP, offer information on the location of the greatest risks of these diseases, and can contribute to the implementation of public health measures aimed at the prevention and improvement of care in these areas. This spatial variability in the distribution of the disease allows us to generate hypotheses about associated factors, based on the current knowledge about the epidemiology of these pathologies and their risk factors. Public health measures should be based on surveillance and the analysis of the existence of associations with possible risk factors, with the further aim of their elimination or at least reduction. This should also include screening and prevention programs aimed at the population at risk, and increased healthcare resources specifically aimed at these pathologies.

Aggregation by small areas, in this case CTs, is used in epidemiological studies [28,29] to highlight geographical differences in the onset of diseases, the existence of distribution patterns or specific aggregates that may be rooted in socioeconomic differences, the distribution of industrial areas or communication routes, etc. The CTs of the study area differed greatly in their size (i.e., large in rural areas and small in urban areas), although the relatively homogeneous reference population reduced the negative effects of using small areas for epidemiological analyses. In addition, their use makes it possible to associate demographic and socioeconomic information with variables for the study of the causes or factors that influence the spatial distribution of diseases. The use of a Bayesian model allowed smoothing the population differences found between the CTs, and attenuated the existence of disparate incidence rates in small but correlated areas [30,31].

The use of the last addresses recorded in the health records―as a source of basic information for the present study―to geocode health events could have made it difficult to interpret the results when these addresses were not the usual residences or, even if they were, the addresses did not correspond to the places where the individuals had the greatest exposure to environmental risks (where they spent most of their time). Similarly, the existence of residential centers for older adults may pose a limitation in the interpretation of the results, because the relationship of such centers in the CT was not included in our study. These factors may affect the rates of some CTs.

## 5. Conclusions

The present study revealed the existence of defined patterns and spatial aggregations in the incidence of AMI and AP, for both men and women, in the central area of Asturias. Our work may serve as a basis for studying the epidemiology of cardiovascular diseases in Asturias, and advance knowledge of the causes and risks of admissions for AMI and AP. Therefore, further studies should be conducted in order to analyze these factors together with environmental and/or socioeconomic aspects, and also taking into account the genetic background.

## Data Availability

The data presented in this study are available on request from the corresponding author. The data are not publicly available due to privacy issues given the small size of the units of study.

## Supplementary Materials

The following are available online at https://www.mdpi.com/article/10.3390/ijerph182312320/s1. Figure S1: The population density of the area of study, Figure S2: Smoothed relative risk of multivariate analysis, Figure S3: Posterior risk probability of multivariate analysis.
