# Supplementary figures and images for: Spatial Distribution of Hospitalizations for Ischemic Heart Diseases in the Central Region of Asturias, Spain
Source: Int J Environ Res Public Health. 2021 Nov 24;18(23):12320. doi: 10.3390/ijerph182312320 (PMC8656975; doi:10.3390/ijerph182312320)

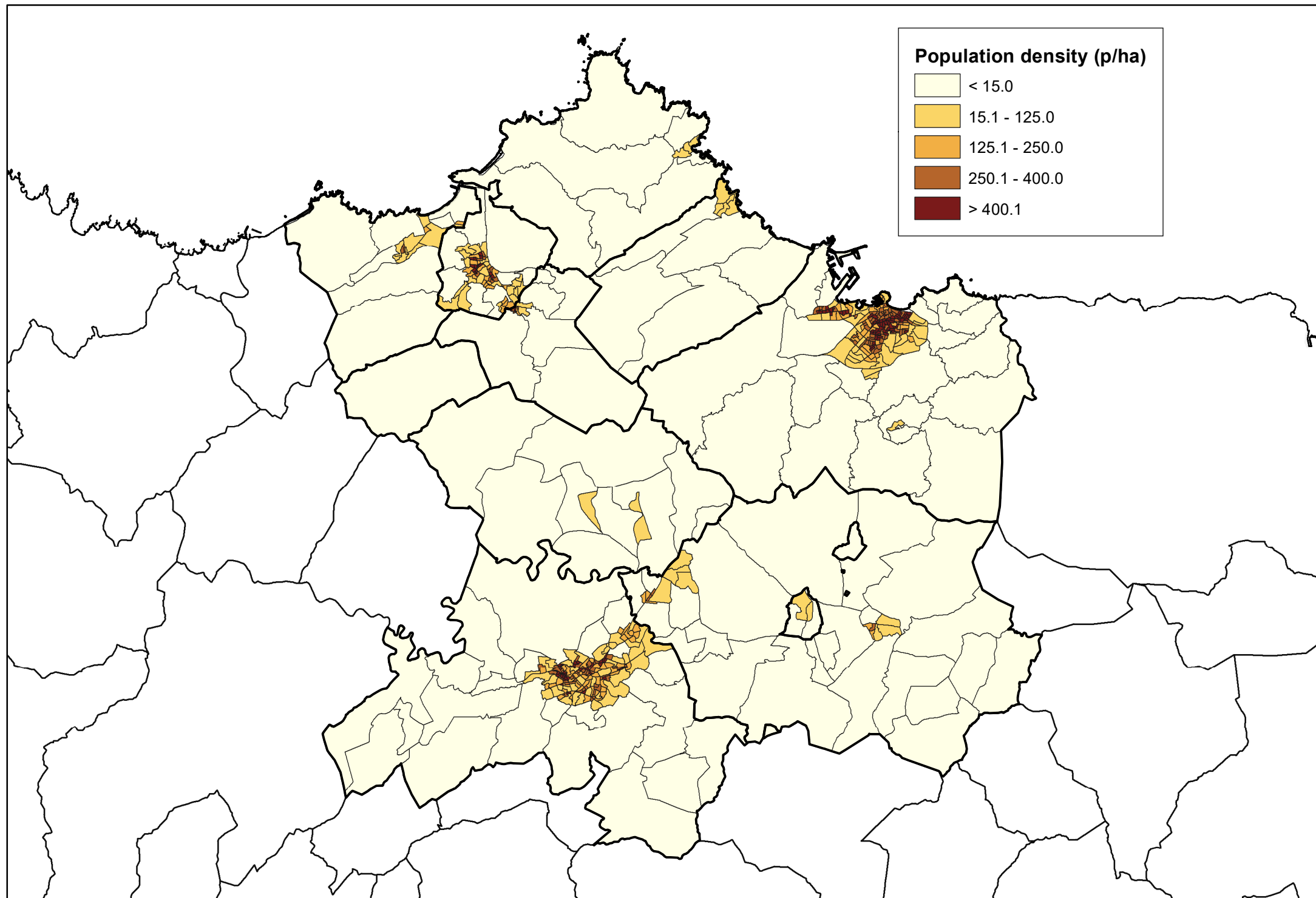

Supplement: Supplementary file 1 [file ijerph-18-12320-s001.zip › FigS1.pdf]

# Smoothed relative risk

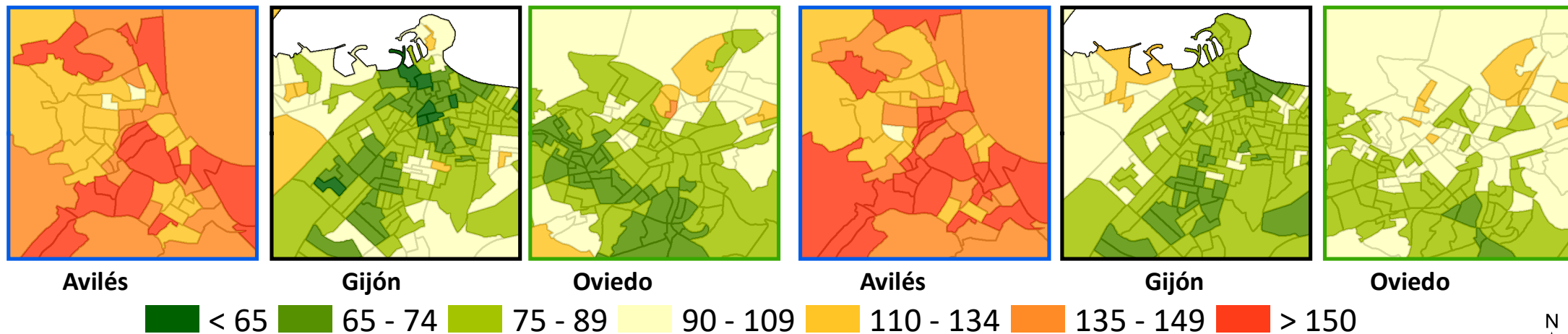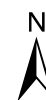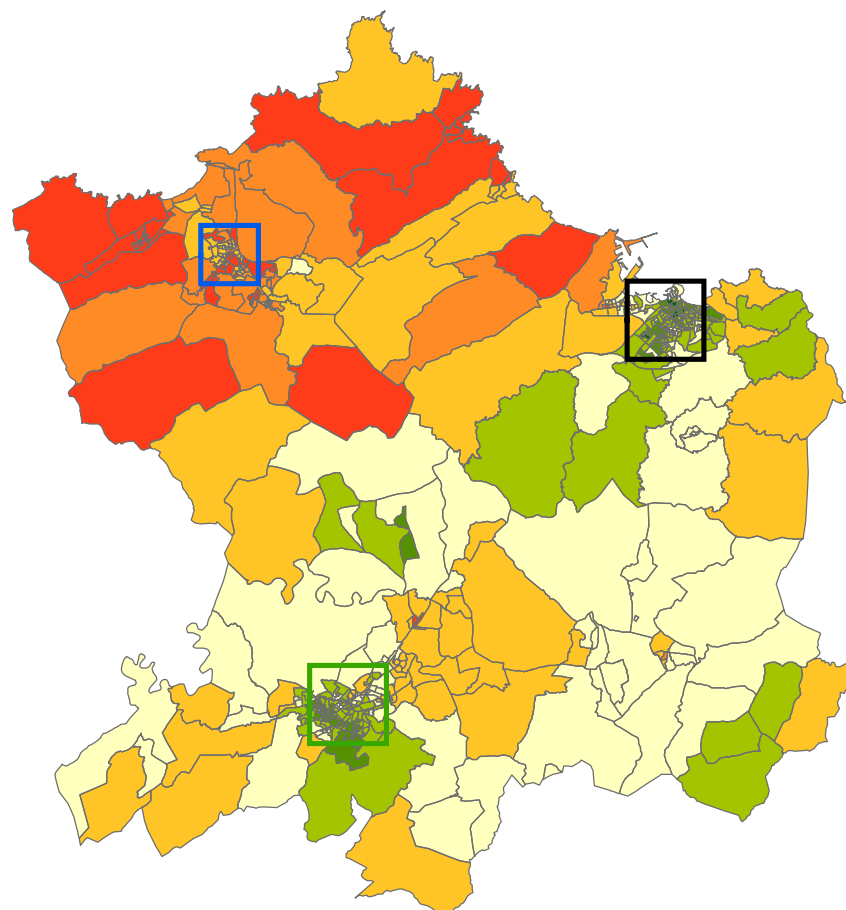

Women

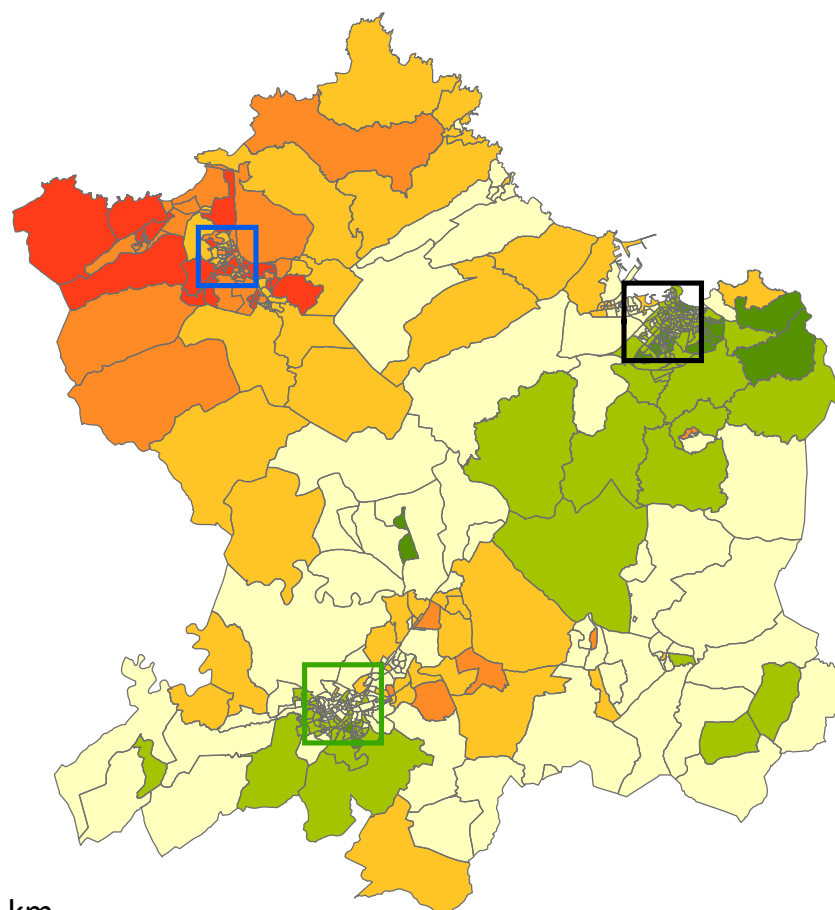

Men

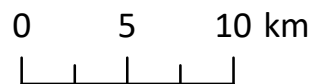

Supplement: Supplementary file 1 [file ijerph-18-12320-s001.zip › FigS2.pdf]

# Posterior risk probability

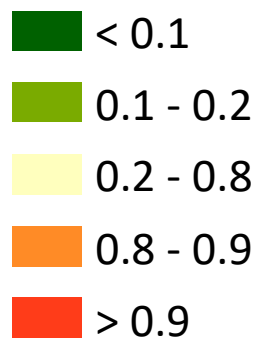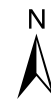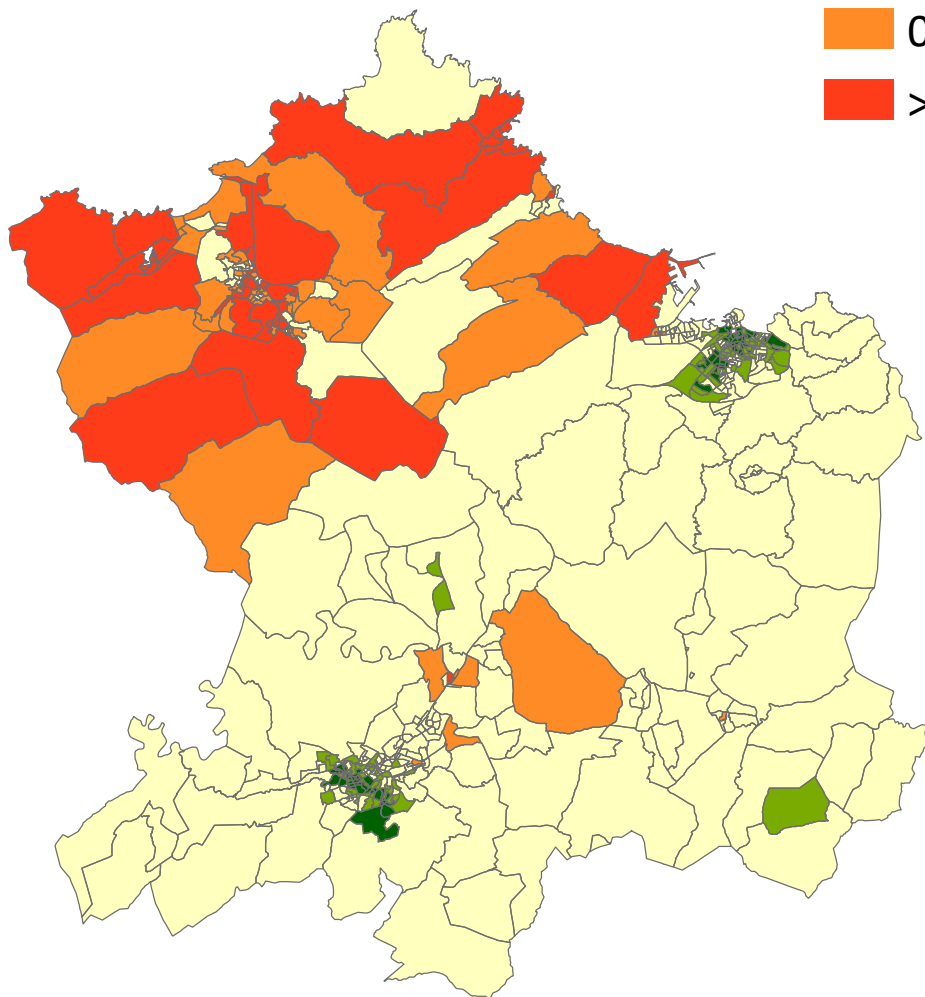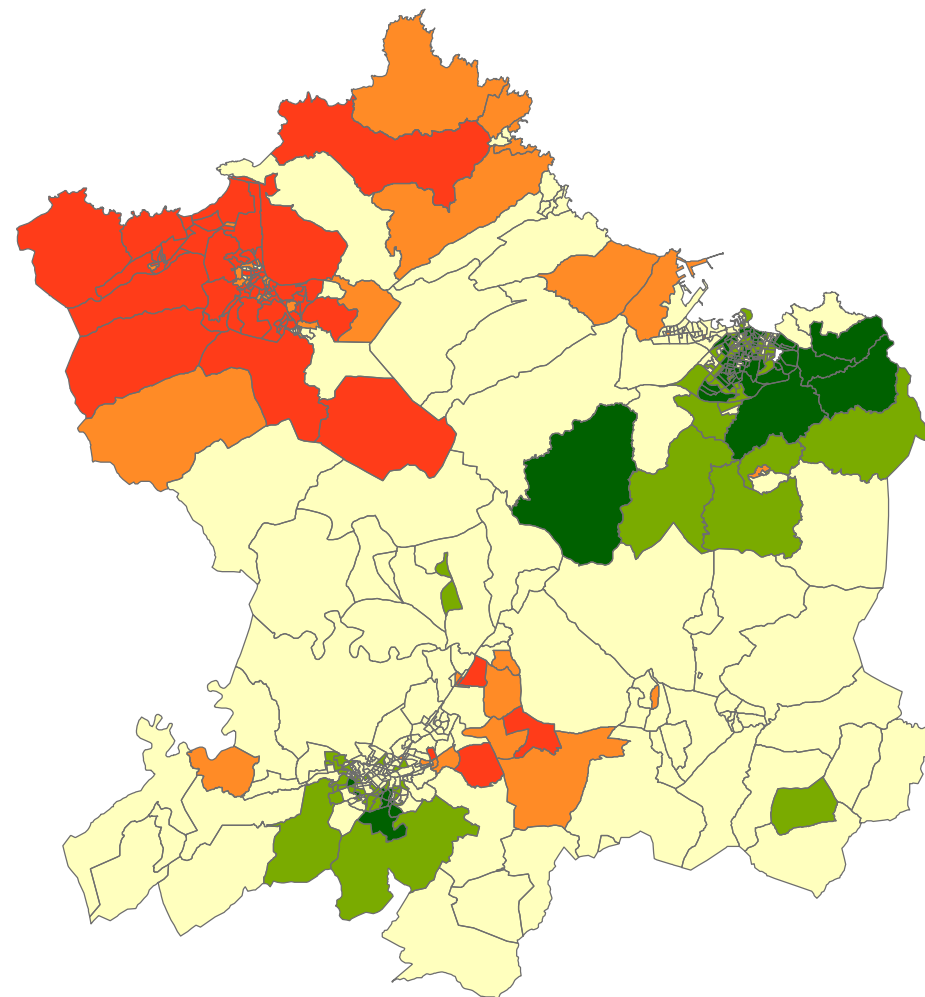

Women

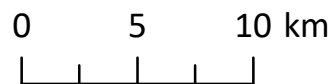

Men

Supplement: Supplementary file 1 [file ijerph-18-12320-s001.zip › FigS3.pdf]
